# Mixed‐stock analysis using Rapture genotyping to evaluate stock‐specific exploitation of a walleye population despite weak genetic structure

**DOI:** 10.1111/eva.13209

**Published:** 2021-03-30

**Authors:** Peter T. Euclide, Tom MacDougall, Jason M. Robinson, Matthew D. Faust, Chris C. Wilson, Kuan‐Yu Chen, Elizabeth A. Marschall, Wesley Larson, Stuart Ludsin

**Affiliations:** ^1^ Wisconsin Cooperative Fishery Research Unit, College of Natural Resources University of Wisconsin‐Stevens Point Stevens Point WI USA; ^2^ Lake Erie Management Unit Ontario Ministry of Natural Resources and Forestry Port Dover ON Canada; ^3^ Lake Erie Fisheries Research Unit New York State Department of Environmental Conservation Dunkirk NY USA; ^4^ Division of Wildlife, Sandusky Fisheries Research Station Ohio Department of Natural Resources Sandusky OH USA; ^5^ Aquatic Research and Monitoring Section Ontario Ministry of Natural Resources and Forestry Peterborough ON Canada; ^6^ Aquatic Ecology Laboratory, Department of Evolution, Ecology, and Organismal Biology The Ohio State University Columbus OH USA; ^7^ U.S. Geological Survey, Wisconsin Cooperative Fishery Research Unit, College of Natural Resources University of Wisconsin‐Stevens Point Stevens Point WI USA; ^8^ Present address: National Oceanic and Atmospheric Administration Ted Stevens Marine Research Institute Juneau AK USA

**Keywords:** genetic stock identification, fisheries management, Great Lakes, Portfolio theory, RAD‐capture, RAD‐seq, stock discrimination

## Abstract

Mixed‐stock analyses using genetic markers have informed fisheries management in cases where strong genetic differentiation occurs among local spawning populations, yet many fisheries are supported by multiple, weakly differentiated stocks. Freshwater fisheries exemplify this problem, with many populations supported by multiple stocks of young evolutionary age and isolated across small spatial scales. Consequently, attempts to conduct genetic mixed‐stock analyses of inland fisheries have often been unsuccessful. Advances in genomic sequencing offer the ability to discriminate among populations with weak population structure, providing the necessary resolution to conduct mixed‐stock assignment among previously indistinguishable stocks. We used genomic data to conduct a mixed‐stock analysis of eastern Lake Erie's commercial and recreational walleye (*Sander vitreus*) fisheries and estimate the relative harvest of weakly differentiated stocks (pairwise *F*_ST_ < 0.01). Using RAD‐capture (Rapture), we sequenced and genotyped individuals from western and eastern basin local spawning stocks at 12,081 loci with 95% reassignment accuracy, which was not possible in the past using microsatellite markers. A baseline assessment of 395 walleye from 11 spawning stocks identified three reporting groups and refined previous assessments of gene flow among walleye stocks. Genetic assignment of 1,075 walleye harvested in eastern Lake Erie's recreational and commercial fisheries indicated that western basin stocks constituted the majority of harvest during the peak walleye fishing season (July–September), whereas eastern basin individuals comprised much of the early season harvest (May–June). Clear spatial structure in harvest composition existed; catches in more easterly sites contained more individuals of eastern basin origin than did more westerly sites. Our study provides important stock contribution estimates for Lake Erie fishery management and demonstrates the utility of genomic data to facilitate mixed‐stock analysis in exploited fish populations having weak population structure or limited existing genetic resources.

## 1 INTRODUCTION

The sustainability of many populations depends on multiple, but often cryptic, breeding groups that are connected by shared habitat and (or) reproductive behaviors (Alves et al., 2010; Cowen et al., 2007; Hilborn et al., 2003). Such complexity contributes to population stability and resilience (Hilborn et al., 2003; Schindler et al., 2010, 2015) but also complicates conservation and management by increasing the number of regulatory units (Cooke et al., 2016). In some cases, populations have been sustainably managed by treating discrete local spawning populations (i.e., stocks) as parts of an overall portfolio of population diversity (Schindler et al., 2015; Waples et al., 2020). Although this idea has primarily been applied to marine species, it has also been suggested for freshwater species (DuFour et al., 2015). However, fisheries management often presumes that assessment information represents a single stock as opposed to multiple stocks, which can lead to unintended overexploitation of local spawning stocks and inappropriate management (Hutchinson, 2008; Li et al., 2015; Stephenson, 1999). The ability to accurately discriminate and identify unique population components (e.g., local spawning stocks) is integral to the conservation and management of populations that fit the portfolio theory model of ecology and evolution, because it provides a means of accounting for variance in stock‐specific productivity (Figge, 2004; Sethi, 2010). However, achieving accurate discrimination among population components can be difficult for species that experience high gene flow at small spatial scales (Martinez et al., 2018) or are of young evolutionary age. Thus, the need exists for methods that can deal with the resultant weak population structure.

For decades, molecular markers have been used to untangle complex migratory behavior (Ruzzante et al., 2006; Seeb & Crane, 1999) and resolve stock‐specific contributions to mixed‐stock fisheries in marine ecosystems (Bernatchez et al., 2017; Milner et al., 2008; Waples et al., 2020). Mixed‐stock assessments have become central to the management of Pacific salmonines (Shaklee et al., 1999) such that the contributions of hundreds of salmon spawning populations are evaluated annually using molecular approaches (Beacham et al., 2020; Dann et al., 2013). Similar practices have become essential to Atlantic Cod *Gadus morhua* management, helping to improve our understanding of seasonal spawning dynamics (Dean et al., 2019) and limit overharvest of less productive stocks (Dahle et al., 2018; Ruzzante et al., 2000).

The fish populations and the fisheries of large freshwater ecosystems, such as the world's Great Lakes, are also often under high fishing pressure (DuFour et al., 2015; Embke et al., 2020; Fluet‐Chouinard et al., 2018) and typically experience more environmental stochasticity than marine ecosystems (Strayer & Dudgeon, 2010) and therefore could benefit from portfolio‐based management. Application of mixed‐stock assessments that use similar techniques as large marine fisheries has been beneficial in large lacustrine fisheries (Andvik et al., 2016; Bott et al., 2009; Potvin & Bernatchez, 2001; Tibihika et al., 2020), especially for species with similar life‐history attributes as marine populations (Ludsin et al., 2014). Such assessments in freshwater ecosystems have been hampered by limited genetic differentiation among spawning stocks (e.g., the North American Great Lakes; Chen, Euclide, et al., 2020; Isermann et al., 2020), highlighting to need for approaches that can detect weak genetic structure.

Fish populations supported by multiple stocks are common in the Laurentian Great Lakes, which have a long history of commercial, recreational, and subsistence fishing (Lynch et al., 2016; Regier & Hartman, 1973). Walleye (*Sander vitreus*) is one of the most ecologically and economically important species in the Great Lakes (Hatch et al., 1987; Ludsin et al., 2014) and has been the focus of many previous stock discrimination research efforts (Chen, Euclide, et al., 2020; Chen et al., 2017; Johnson et al., 2004; Stepien et al., 2015). Lake Erie's walleye population is the largest of the five Laurentian Great Lakes and is supported by multiple spatially and biologically discrete stocks (Chen, Ludsin, et al., 2020; Stepien et al., 2012; Stepien & Faber, 1998; Zhao et al., 2011). Most of the lake's walleye production occurs in the western basin where tributary and open‐lake spawning stocks of varying productivity exist (DuFour et al., 2015; Fraker et al., 2015). Individuals from these stocks move throughout Lake Erie during nonspawning periods in search of preferred habitat (Kershner et al., 1999; Raby et al., 2018), where they intermix with walleye from smaller spawning stocks in the central and eastern basins (Matley et al., 2020; Vandergoot & Brenden, 2014; Zhao et al., 2011). Migration of individuals from western basin stocks into Lake Erie's eastern basin is predicted to have a disproportionate influence on local commercial and recreational fisheries because of presumed differences in population productivity and abundance (Zhao et al., 2011). However, the exact degree to which individuals from western versus eastern spawning stocks are harvested by recreational and commercial fisheries in a year or season is unclear. Further, efforts to discriminate among western and eastern basin stocks to facilitate mixed‐stock assessments using biological markers have been largely unsuccessful (Chen et al., 2017; Hedges, 2002; Johnson et al., 2004; Riley & Carline, 1982). These needs, in turn, limit management options in Lake Erie (MDF, JMR, TMM).

A recent study using thousands of single nucleotide polymorphisms (SNPs) genotyped with restriction sited associated DNA sequencing (RAD‐seq) showed high‐accuracy reassignment of walleye to the basin of origin, suggesting potential use high‐throughput sequencing for mixed‐stock assessments (Chen, Euclide, et al., 2020). Building on this study, we developed a Rapture panel containing thousands of genetic markers to conduct a mixed‐stock analysis of walleye harvest in Lake Erie's eastern basin. Specifically, our objectives were to 1) quantify the relative contributions of western and eastern basin walleye spawning populations to commercial and recreational harvest within the eastern basin, and 2) determine whether these contributions varied spatiotemporally. We hypothesized that individuals spawned in the western basin would comprise the majority of harvest for both types of fisheries, but that harvest composition would vary spatially and temporally owing to annual seasonal migrations (Kershner et al., 1999; Raby et al., 2018). In testing this hypothesis, we ultimately demonstrate the ability of genomic data to resolve genetic stock structure and facilitate mixed‐stock analysis in ecosystems with weak population structure, to the benefit of fisheries management.

## 2 MATERIALS AND METHODS

### 2.1 Study system and sample collection

**FIGURE 1:**
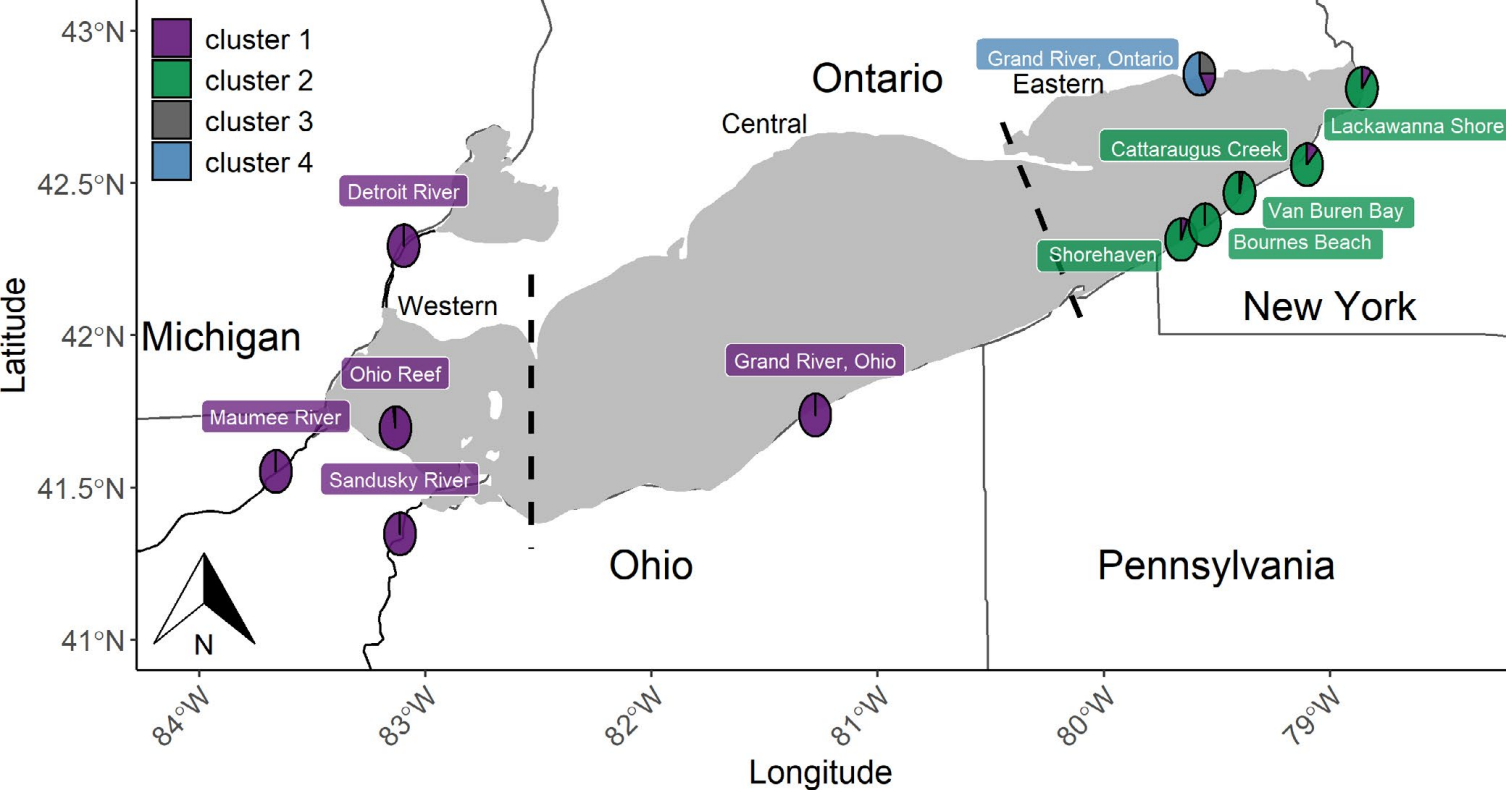
Map of collection locations of walleye (*N* = 397) within Lake Erie that were used to develop the baseline genotypes for Lake Erie spawning stocks. The background color of each spawning location indicates the final reporting group into which that local spawning stock was grouped based on assignments generated from discriminant analysis of principal components (DAPC). This analysis used Bayesian Information Criterion to group walleye taken from these known spawning locations during 2012–2018 spawning seasons (Table 1) into the four clusters. Pie charts display the posterior probability of each DAPC cluster proportion averaged across all individuals collected at that spawning location (color scheme is identical to that used for spawning locations). Lake basins (western, central, and eastern) are delineated by dashed black lines

Lake Erie has a surface area of ~26,000 km^2^ and is composed of three basins. The western basin is shallowest with most areas 3–7 m in depth. The central basin is deeper (15–18 m deep), and the eastern basin is the deepest (15–25 m) with the deepest point in the lake being 65 m (Holcombe et al., 2003). Four large walleye stocks exist in the western basin. The largest stock spawns on open‐lake reefs, whereas the others spawn in three large tributaries (Maumee, Sandusky, and Detroit rivers; DuFour et al., 2015; Fraker et al., 2015; Figure 1). Spawning occurs in the central basin on nearshore reefs and in the Grand River, Ohio (Stepien et al., 2018). Although the contributions of their resulting offspring to the lake‐wide fishery remains unknown, we suspect them to be less than the western basin stocks because their spawning populations are smaller. More spawning stocks exist in the eastern basin, where spawning aggregations have been reported in small New York tributaries and nearshore reefs along the south‐eastern shore, as well as in Ontario's Grand River on the northern shore of the eastern basin (Zhao et al., 2011; Figure 1).

Walleye samples of both known origin and unknown origin were collected by the Ohio Department of Natural Resources—Division of Wildlife (western basin), New York State Department of Environmental Conservation (south shore of the eastern basin), and Ontario Ministry of Natural Resources and Forestry (north shore of the eastern basin). Fin clips were taken from all individuals and preserved in 95% ethanol until processing for molecular work. We extracted DNA from all fin clips using Qiagen DNEasy 96 kits (Qiagen).

Given that spawning‐site fidelity for Lake Erie walleye is high (70%–98%; Chen, Ludsin, et al., 2020; Hayden et al., 2018) and all individuals used for initial panel development (*N* = 96) and development of our baseline classification functions (*N* = 397) were (1) collected at known spawning locations that are spatially segregated from nonspawning and (2) determined to be mature and in spawning condition (i.e., males were ripe and females were gravid but hard or “spent”), we are confident that all individuals of known origin were spawning at or had recently spawned at that their collection locations (Table 1; Figure 1). Individuals of unknown origin (*N* = 1274) were sampled during the spring, summer, and fall of the 2016–2018 fishing seasons (Table 2). The total length (nearest 1 mm) of each individual, as well as its harvest location (nearest creel grid location), was collected by agency personnel (e.g., creel agents) at boat ramps and docks (Table S1). To maximize our ability to quantify seasonal variation in stock‐specific harvest in eastern Lake Erie's fisheries, the majority (*N* = 1021, 80%) of the unknown origin walleye used in our mixed‐stock analyses were from the 2017 harvest, with samples chosen in proportion to their proportional harvest during May through December (Table 2). We also selected 130 samples from the July 2016 commercial harvest and 122 from the July 2018 recreational harvest to investigate interannual variation during the season of peak harvest (Table 2).

**TABLE 1:** Walleye samples collected and genotyped for Rapture panel development and identification of reporting groups in mixed‐stock analysis of the recreational and commercial harvests in the eastern Lake Erie during 2016–2018

Spawning stock	Year(s)	Stock location	Reporting group	*N*	*H*_O_	*H*_E_	AR	*F*_IS_
Panel development								
Maumee River	2012–2014	Western	West/central basin	13	0.31	0.29	1.67	−0.08
Sandusky River	2012–2014	Western	West/central basin	18	0.30	0.30	1.67	−0.03
Detroit River	2012–2014	Western	West/central basin	13	0.29	0.31	1.66	−0.08
Ohio Reefs	2012–2014	Western	West/central basin	26	0.29	0.29	1.67	−0.01
Ohio Grand River	2012–2014	Central	West/central basin	13	0.29	0.28	1.67	−0.03
Van Buren Bay	2012–2014	Eastern	East basin	13	0.28	0.29	1.66	−0.00
Baseline samples (for reporting group classification)								
Maumee River	2017, 2018	Western	West/central basin	36	0.32	0.28	2.28	−0.09
Sandusky River	2017, 2018	Western	West/central basin	36	0.31	0.29	2.33	−0.04
Detroit River	2017, 2018	Western	West/central basin	36	0.30	0.29	2.32	−0.02
Ohio Reef Complex	2017	Western	West/central basin	36	0.31	0.29	2.30	−0.06
Ohio Grand River	2012–2014	Central	West/central basin	13	0.30	0.29	2.38	−0.03
Van Buren Bay	2017, 2018	Eastern	East basin	38	0.29	0.29	2.32	−0.01
Cattaraugus Creek	2017, 2018	Eastern	East basin	18	0.29	0.28	2.32	−0.02
Lackawanna Shore	2017	Eastern	East basin	24	0.30	0.28	2.29	−0.03
Bournes Beach	2018	Eastern	East basin	14	0.33	0.28	2.25	−0.15
Shorehaven	2017	Eastern	East basin	50	0.29	0.29	2.32	0.00
Ontario Grand River	2014, 2016	Eastern	Ontario Grand River	96	0.29	0.29	2.29	0.03

Note

Columns show total sample size (*N*) and genetic diversity estimates including observed (*H*_O_) and expected (*H*_E_) heterozygosity, allelic richness (AR), and inbreeding coefficient (*F*_IS_) for final Rapture panel single nucleotide polymorphism (SNP) genotypes. These SNPs were identified during initial RAD‐sequencing for panel development. Afterward, microhaplotypes containing these SNPs were genotyped using a Rapture panel developed from the 395 baseline individuals and used to identify reporting groups (note the higher AR of microhaplotype loci than SNP loci in Baseline individuals). Panel Development samples generated the initial RAD‐sequencing data that were subsequently used to develop the Rapture panel. This panel, in turn, was used to describe the Baseline population structure in Lake Erie and assign individuals of unknown origin.

**TABLE 2:** Number of walleye of unknown origin collected in eastern Lake Erie's commercial and recreational fisheries that were genotyped (*N* total), successfully assigned (classified) to one of three natal areas (i.e., spawning groups: western/central basin, eastern basin, or Ontario Grand River; *N* assigned), and assigned to a noneastern basin spawning group (*N* assigned to west/central basin)

Year	Data source	Month	Fishery	*N* total	*N* assigned	*N* assigned to west/central basin	% Assigned to west/central basin
2016	NY	June	Recreational	7	5	1	20
2016	NY	July	Recreational	115	98	20	20
2016	NY	August	Recreational	7	6	6	100
			2016 Totals	129	109	27	25
2017	NY	May	Recreational	28	26	1	4
2017	NY	June	Recreational	118	101	23	23
2017	NY	July	Recreational	138	120	65	54
2017	NY	Aug.	Recreational	70	64	50	78
2017	NY	Sep.	Recreational	126	114	91	80
2017	NY	Oct.	Recreational	35	34	22	65
2017	ON	May	Commercial	9	9	1	11
2017	ON	June	Commercial	117	98	39	40
2017	ON	July	Commercial	124	106	41	39
2017	ON	Aug.	Commercial	40	34	16	47
2017	ON	Sep.	Commercial	125	105	74	70
2017	ON	Oct.	Commercial	30	30	7	23
2017	ON	Nov.	Commercial	30	28	26	93
2017	ON	Dec.	Commercial	30	27	27	100
			2017 Totals	1020	896	483	54
2018	ON	July	Commercial	110	62	56	90
2018	ON	August	Commercial	12	5	4	80
			2018 Totals	122	67	60	90

Note

To facilitate interpretation, the percentage of fish originating outside of the eastern basin (i.e., west/central basin) is also reported. Assignment to a reporting groups is categorized by the harvest year (2016, 2017, or 2018), sample source (either New York State Department of Environmental Conservation [NY] or the Ontario Ministry of Natural Resources and Forestry [ON]), month of harvest, and fishery type. No individuals were assigned to the Grand River, Ontario reporting group; thus, the remainder of walleye not assigned to the west/central basin reporting group originated from eastern basin (non‐Grand River, ON) spawning locations. Individuals that could not be assigned to a natal source had >50% missing genotypes and were removed from analysis.

### 2.2 Rapture panel development

We developed a Rapture bait panel (Ali et al., 2016) to reduce cost and increase the power and consistency of genetic stock identification. Loci containing SNPs and microhaplotypes (short DNA fragments containing multiple polymorphic SNPs) were identified by conducting RAD‐sequencing on 96 walleye collected at spawning sites across the lake (Table 1). A single RAD‐seq library was prepared using *SbfI* enzyme and the standard library preparation approach outlined in Ali et al. (2016) and detailed in Ackiss et al. (2020). Samples were sequenced using paired‐end 150 BP sequencing on an Illumina HiSeq4000 at NovoGene. Sequencing produced a total of 61,350,730 retained reads and an average effective per‐sample coverage of 11.4 (standard deviation = 4.0). Loci were then assembled *de novo* in STACKS v. 2.0 (Catchen et al., 2011; Rochette et al., 2019) and a catalog of all putative loci was created in *cstacks* using data from all 96 individuals. Samples were demultiplexed using *process_radtags* (‐e sbfI ‐i gzfastq ‐c ‐q ‐r ‐‐filter_illumina –bestrad), assembled *de novo* in *ustacks* (‐‐disable gapped, ‐m3, ‐M 3, ‐H, ‐‐max_locus_stacks 4, ‐‐model_type bounded, ‐‐bound_high 0.05), matched in *sstacks* (‐‐disable gapped), converted to bam files using *tsv2bam*, and genotyped in *gstacks* resulting in 263,723 putative SNPs and 43,884 loci. Finally, genotypes were called for only SNPs with a minor allele count greater than three (‐‐mac 3) to avoid potential sequencing error while maintaining potentially informative rare alleles (O'Leary et al., 2018). We then removed putative paralogs identified in HDPlot (McKinney et al., 2017), as well as loci with minor allele frequencies <0.01, heterozygosity <0.05, and genotype rate <50%.

Sequence data for the remaining 14,418 loci that met quality standards were sent to Arbor Biosciences for bait development (custom oligonucleotides that help to isolate desired genomic regions). Arbor Biosciences conducted additional quality filters, including a blast alignment to the yellow perch (*Perca flavescens*) genome (Feron et al., 2019) that removed an additional 2337 loci, and then synthesized two baits per‐locus to create a final panel consisting of 12,081 loci (24,162 unique baits). STACKS 2 catalog files and a fasta file for all 12,081 baited loci can be found on Dryad (https://doi.org/10.5061/dryad.4b8gthtb2).

### 2.3 Rapture sequencing

RAD‐seq libraries for the 397 walleye used to establish our baseline (known origin) genetics signatures of population structure were prepared identically as individuals used to develop the panel, with the subsequent bait capture being conducted for each library following the myBaits protocol v.4.01(https://arborbiosci.com/mybaits‐manual/) with minor modifications. In short, RAD‐seq libraries were hybridized with the bait mixture for 16 h at 65°C and then amplified using 10 PCR cycles, universal primers, and an annealing temperature of 56°C. Final Rapture libraries were purified with a 1X Ampure bead solution and submitted for sequencing on ½ of a S4 NovaSeq lane at NovoGene. Data were processed using STACKS v.2.3 (Rochette et al., 2019) with identical parameters and catalog as Rapture panel development. Loci were then filtered using a locus‐specific whitelist that included only the 12,081 loci in the Rapture panel. Microhaplotypes at each locus were identified in the *populations* step of STACKS and used for all downstream analysis. To ensure that the SNPs used in microhaplotype genotypes were not the result of sequencing error, each SNP had to be genotyped in at least 80% of individuals (both baseline and mixed‐stock) and have a minor allele count of three or more.

Rapture libraries for the mixed‐stock assignment of the 1274 individuals of unknown origin were constructed using identical procedures as the baseline samples with one exception; to reduce the number of bait capture reactions necessary to genotype mixed‐stock samples, DNA from two RAD‐seq libraries were included in each bait capture reaction. All mixed‐stock Rapture libraries were pooled and sequenced on four Illumina HighSeq4000 lanes at NovoGene. Sequence data were processed in STACKS v.2.3 using identical procedures and filters as the baseline samples with the exception of individual genotype rate, which was reduced to 50% because assignPOP v.1.1.8 R package (Chen et al., 2018) indicated similar reassignment accuracy between 100% and 50% retained loci tests.

### 2.4 Baseline population structure and identification of reporting groups

*F*‐statistics and discriminant analysis of principal components (DAPC; Jombart et al., 2010) were used to describe overall patterns of genetic structure in Lake Erie and to identify putative reporting groups for reassignment tests to determine classification accuracies. Observed and expected heterozygosity, inbreeding coefficient, and pairwise Weir and Cockerham (1984) *F*_ST_ were estimated in the DiveRsity v.1.1.9 R package (Keenan et al., 2013; R Core Team, 2019). We used DAPC to identify putative clusters of spawning sites that could be combined into reporting groups for assignment of individuals of unknown origin. We ran DAPC using the ADEGENET v.2.1.2 R package, first with individuals grouped by spawning stock and then with individuals grouped into four clusters identified with the *find*.*clusters* function (Jombart, 2008). To avoid model over‐fitting, the optimal number of principal components necessary to explain among‐group variance was identified in ADEGENET using the *optim*.*a*.*score* function (Jombart, 2008). Finally, we used AMOVA and permutation tests of significance implemented in the poppr v.2.8.3 R package (Kamvar et al., 2014) to determine how much variance was explained when sites were grouped by putative reporting groups.

We tested reassignment accuracy of putative reporting groups in the assignPOP v.1.1.8 R package (Chen et al., 2018). Assignment tests were performed using Monte‐Carlo cross‐validation in which individuals were randomly resampled as a training set, using remaining individuals as a test (holdout) set to avoid upwardly biased test results (Anderson, 2010; Waples, 2010). Specifically, we chose three proportions (0.5, 0.7, and 0.9) of individuals from each reporting group and used either half (chosen randomly) or all loci as training data (*N* = 6 total combinations) to perform the assignment test. Each combination of training data and the test dataset were iterated 30 times for a total of 180 assignment tests. This procedure allowed us to evaluate variation in assignment accuracy and how different proportions of training individuals influenced the assignment results.

The assignPOP predictive models were built using a support vector machine (with linear kernel and parameter cost = 1), which has been shown to generate higher assignment accuracies than other models (i.e., LDA, naiveBayes, decision tree, and random forest; Chen et al., 2018) and had the highest accuracy in our preliminary runs. We ran the reassignment test twice: once with every spawning stock kept separate and once with stocks clustered into putative reporting groups identified with DAPC and pairwise *F*_ST_. Because individual assignments can sometimes bias mixture results, we compared assignment accuracy identified in assignPOP with 100% mixture assignment tests conducted in the rubias R package v.0.3.0 (Moran & Anderson, 2019). Assignment accuracy of 100% mixtures for all reporting groups was estimated using a leave‐one‐out approach, 25 replicates, and a mixture size of 100. All presented graphics were constructed in R primarily using the ggplot 3.3.0 (Wickham, 2009), ggpubr v.0.2.5 (Kassambara, 2020), ggsci v.2.9 (Xiao, 2018), and the sf v.0.7‐7 (Pebesma, 2018), and scatterpie v.0.1.4 (Yu, 2019) packages.

### 2.5 Mixed‐stock assignment of harvested walleye

Once all harvested individuals were genotyped, each was assigned to a reporting group (i.e., natal sources identified during baseline analysis) using the microhaplotype genotypes and the SVM parameter in the *assign*.*x* function of assignPOP (Chen et al., 2018). As an additional estimate of mixed‐stock proportion in 2017, the total stock mixture was estimated in rubias with identical reporting groups and reference as assignPOP and bootstrapped proportions (100 bootstraps). As confirmation of individual assignments, we also used rubias to estimate the stock mixture of all individuals assigned to each reporting group. Individual assignments were presumed to be correct as long as the genetic mixture of individuals in each reporting group exceeded 90%.

### 2.6 Stock‐specific harvest dynamics

To determine how spawning stock contributions to the eastern basin fisheries varied through space and time, all walleye that could be successfully assigned to one of our reporting groups were summarized by year, month, location (harvest grid centroid), and fishery type (recreational or commercial). For walleye sampled during 2017, the proportion of individuals assigned to each reporting group was calculated by dividing the number of individuals assigned to a particular reporting group by the total number assigned for a particular month, location, or fishery type. Because some locations contained a small number of assigned individuals during certain months (e.g., June and August 2016), only month/location samples with greater than six successfully assigned walleye were included (*N* = 48 grid‐by‐month samples; Table 2).

Using the proportion of walleye assigned to the west/central basin reporting group (see Section 3) as our response variable, we conducted analysis of variance (ANOVA) tests on linear models using only 2017 data with fishery type, month, and longitude as predictor variables. We used a grid's longitude instead of its grid identification number to help identify west‐to‐east gradients in harvest. Estimates of stock proportion were also extrapolated to actual estimates of harvest (number of fish) for each fishery type, location, and month during 2017, using harvest estimates provided by Lake Erie's Walleye Task Group (2018).

To describe interannual variation in stock‐specific harvest, we compared the proportional contributions of each reporting group during July 2016–2018. Only samples collected during July 2016–2018 were used because July is consistently one of the peak months of walleye harvest in Lake Erie (Walleye Task Group, 2018) and we had a high number of available samples for all three years. These data were compared by calculating the proportion of individuals assigned to each reporting group relative to the total fish assigned to any reporting group that year. Because only a single month of samples was genotyped during 2016 and 2018, comparisons should be only considered as point estimates of proportional harvest.

Given that the relative proportions of individuals assigned to a particular reporting group throughout the season may be predicted by more than a single variable, we used an information theoretic approach to identify the most parsimonious predictive model of the proportional contribution from the western/central basin reporting group. Specifically, we used Akaike's Information Criterion for small sample sizes (AIC_c_; Burnham & Anderson, 2004) to evaluate seven generalized linear models (GLMs) consisting of different combinations of predictor variables, including the type of fishery (commercial or recreational), average total length of individuals sampled, latitude of capture, longitude of capture, and month of harvest. All explanatory variables were first run independently against the response variable (proportion of walleye assigned to the west/central basin reporting group), with any variable not found to be significant (ANOVA; *α* = 0.05) being removed from future models. To test the relationship between fishery type and harvest proportion type was converted to a numeric dummy variable (0 or 1). Once a subset of variables that were all independently significant was identified, models containing all possible combinations of these variables were built, and the best model was chosen based on the approach outlined in Symonds and Moussalli (2011). We did not include interaction terms in these models because we did not have any a priori predictions to justify them. In short, each model was fit in R using the *glm* function using default settings (Gaussian error structure) and then ranked based on AIC_c_ using the AICcmodavg v.2.3 R package whereby the model with the lowest AIC_c_ was considered the most parsimonious. Finally, the Evidence Ratio (ER):

$$\text{ER}=\frac{\exp\left(‐\frac{1}{2}\Delta_{\text{best}\;}\right)}{\exp\left(‐\frac{1}{2}\Delta_{n}\right)}$$

was calculated in the qpcR v.1.4‐1 R package where $\Delta_{\text{best}}$ equals 0 as a measure of how much more likely the best model was than model *n* whereby a low evidence ratio indicates that a model is more similar to the best model (Mazerolle, 2020; Spiess, 2018).

## 3 RESULTS

### 3.1 Baseline population structure and identification of reporting groups

Sequencing of 397 walleye collected from known Lake Erie spawning sites during the spawning season produced a total of 1,953,890,992 reads and an average effective per‐sample coverage of 124.2 (standard deviation = 44.2). Of the 12,091 baited SNP loci identified, 8482 loci passed our genotyping criterion (present in ≥80% of individuals) and allele count filter (minor allele count >3). Microhaplotypes at each locus had an average of 3.8 alleles, ranging 1 to 21 (Table S2). Two individuals contained genotypes at fewer than 70% of loci and thus were removed. As a result, the baseline population structure analysis was conducted using 395 individuals genotyped at 92% of retained loci.

**FIGURE 2:**
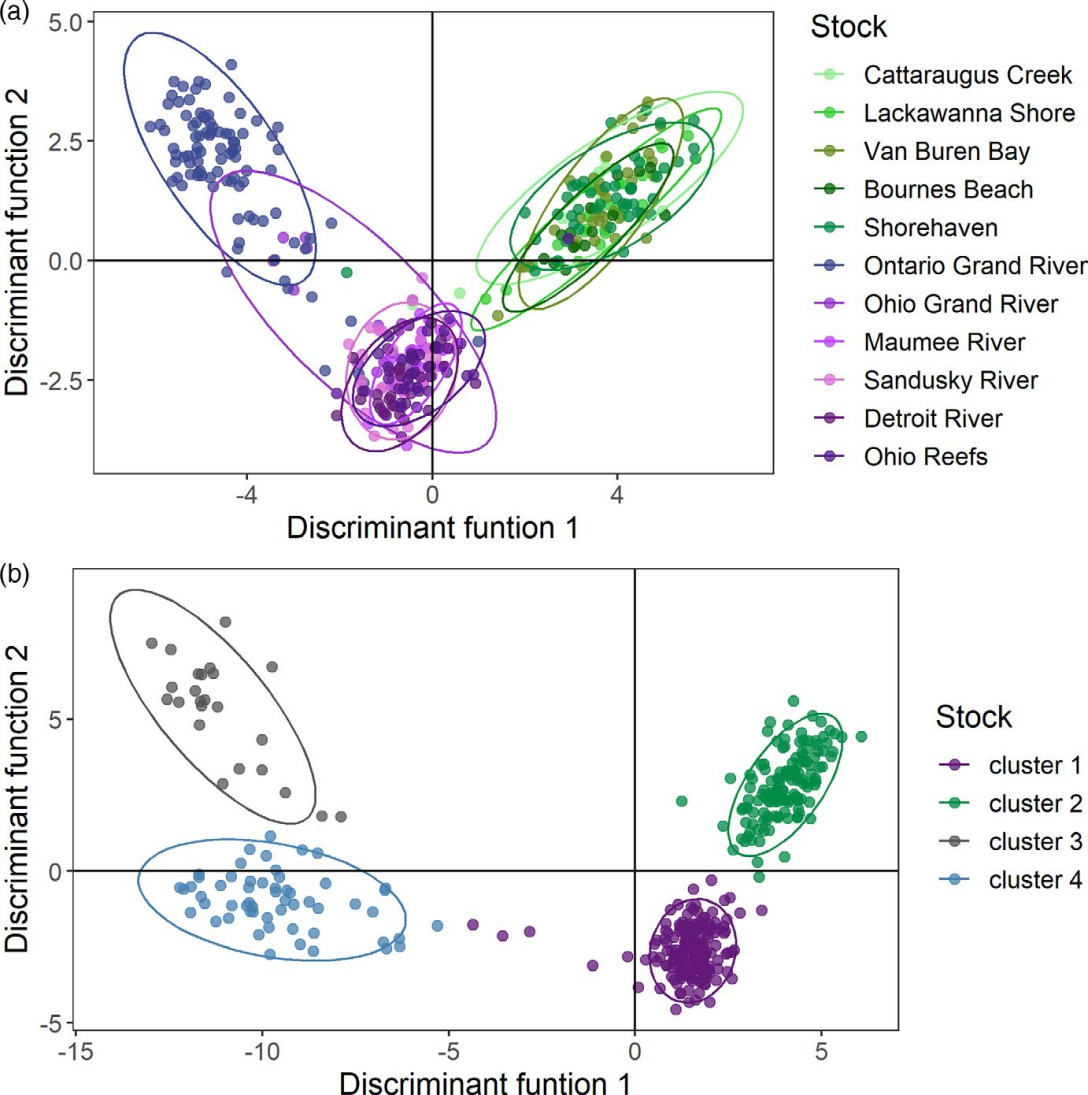
Discriminant analysis of principal components (DAPC) of walleye (individual points) collected on Lake Erie spawning grounds during the 2012–2018 spawning seasons. Individuals are grouped by local spawning stock (a) and or by the four most parsimonious clusters based on Bayesian Information Criterion (b). Ellipses show the 95% confidence interval around each group. Colors in A correspond approximately to the reporting groups shown in Figure 1: west/central basin = purples; eastern basin = greens; and Grand River, Ontario = blue

Three to four genetically similar clusters of spawning stocks were identified with DAPC (Figure 2) and Pairwise *F*_ST_ (Table 2). When coded by spawning site, DAPC grouped stocks into three clusters, two corresponding to the eastern and western basins and one corresponding to individuals from the Ontario Grand River (Figure 1a). When individuals were grouped without a priori site information, four clusters were identified as the most parsimonious groupings of individuals based on Bayesian Information Criterion (Figure 2b). The first of these clusters contained 142 out of 143 of the western basin and 13 out of 13 central basin walleye. The second cluster contained 139 out of 142 individuals collected from five of the six eastern basin stocks sampled (all but the Grand River, Ontario). Finally, the third and fourth clusters contained 24 and 56 individuals out of 96 Ontario's Grand River, individuals respectively while the remaining 16 individuals from Ontario's Grand River were genetically similar to west/central basin individuals and assigned to cluster 1. A similar pattern was apparent with pairwise *F*_ST_ (Table 3) whereby pairwise *F*_ST_ was highest between Ontario's Grand River and all other sites in the eastern basin and all of the western basin sites (average *F*_ST_ = 0.032). Importantly, however, *F*_ST_ values were higher between basins (west/central basin stocks vs. non‐Grand River eastern basin stocks; mean pairwise *F*_ST_ = 0.007) than within them (mean pairwise *F*_ST_ = 0.003).

**TABLE 3:** Pairwise genetic differences (*F*_ST_; Weir and Cockerham, 1984) of walleye collected at different Lake Erie spawning locations during 2014–2017 (bottom of diagonal) and bootstrapped 95% confidence intervals (top of diagonal)

Spawning stock	Maumee River	Sandusky River	Detroit River	Ohio Reef complex	Grand River (Ohio)	Van Buren Bay	Cattaraugus Creek	Lackawanna Shore	Bournes Beach	Shorehaven	Grand River (ON)
Maumee R.	0	−0.0018–0.0091	−0.0006–0.0092	−0.0016–0.0095	−0.0064–0.0228	0.0025–0.0126	−0.0001–0.0192	−0.0002–0.0154	0.0032–0.0259	0.0037–0.0116	0.0231–0.0344
Sandusky R.	0.0030	0	−0.0026–0.0064	−0.0023–0.0064	−0.0096–0.0198	0.0007–0.0104	−0.0009–0.0194	−0.0015–0.0125	0.0011–0.0207	0.0022–0.0108	0.0212–0.0314
Detroit R.	0.0039	0.0018	0	−0.0025–0.0058	−0.0093–0.0177	0.0021–0.0092	−0.0019–0.0173	−0.0015–0.0125	0.0022–0.0213	0.0024–0.0088	0.0225–0.0317
Ohio Reefs	0.0034	0.0022	0.0017	0	−0.0073–0.0198	0.0015–0.0102	−0.0012–0.0182	−0.0014–0.0123	0.0017–0.0224	0.0023–0.0091	0.0239–0.0321
Grand R. (OH)	0.0060	0.0031	0.0019	0.0044	0	−0.0084–0.0241	−0.0095–0.0267	−0.0104–0.0234	−0.0046–0.0340	−0.0062–0.0232	0.0100–0.0403
Van Buren Bay	0.0069	0.0056	0.0054	0.0055	0.0054	0	−0.0068–0.0140	−0.0068–0.0087	−0.0037–0.0174	−0.0024–0.0043	0.0257–0.0370
Cattaraugus Cr.	0.0081	0.0066	0.0061	0.0070	0.0054	0.0012	0	−0.0093–0.0180	−0.0048–0.0206	−0.0059–0.0131	0.0223–0.0433
Lackawanna Sh.	0.0069	0.0052	0.0055	0.0052	0.0058	4.00E−04	0.0015	0	−0.0037–0.0177	−0.0045–0.0085	0.0243–0.0406
Bournes Beach	0.0118	0.0107	0.0098	0.0107	0.0126	0.0045	0.0068	0.0055	0	−0.0036–0.0164	0.0256–0.0483
Shorehaven	0.0070	0.0057	0.0056	0.0055	0.0058	6.00E−04	0.0011	5.00E−04	0.005	0	0.0262–0.0355
Grand R. (ON)	0.0279	0.0260	0.0274	0.0277	0.0246	0.0312	0.0309	0.0315	0.0342	0.0306	0

Note

Individuals from these local spawning stocks were used in the baseline assessment of population structure of Lake Erie and used to define reporting groups for mixed‐stock analysis.

**FIGURE 3:**
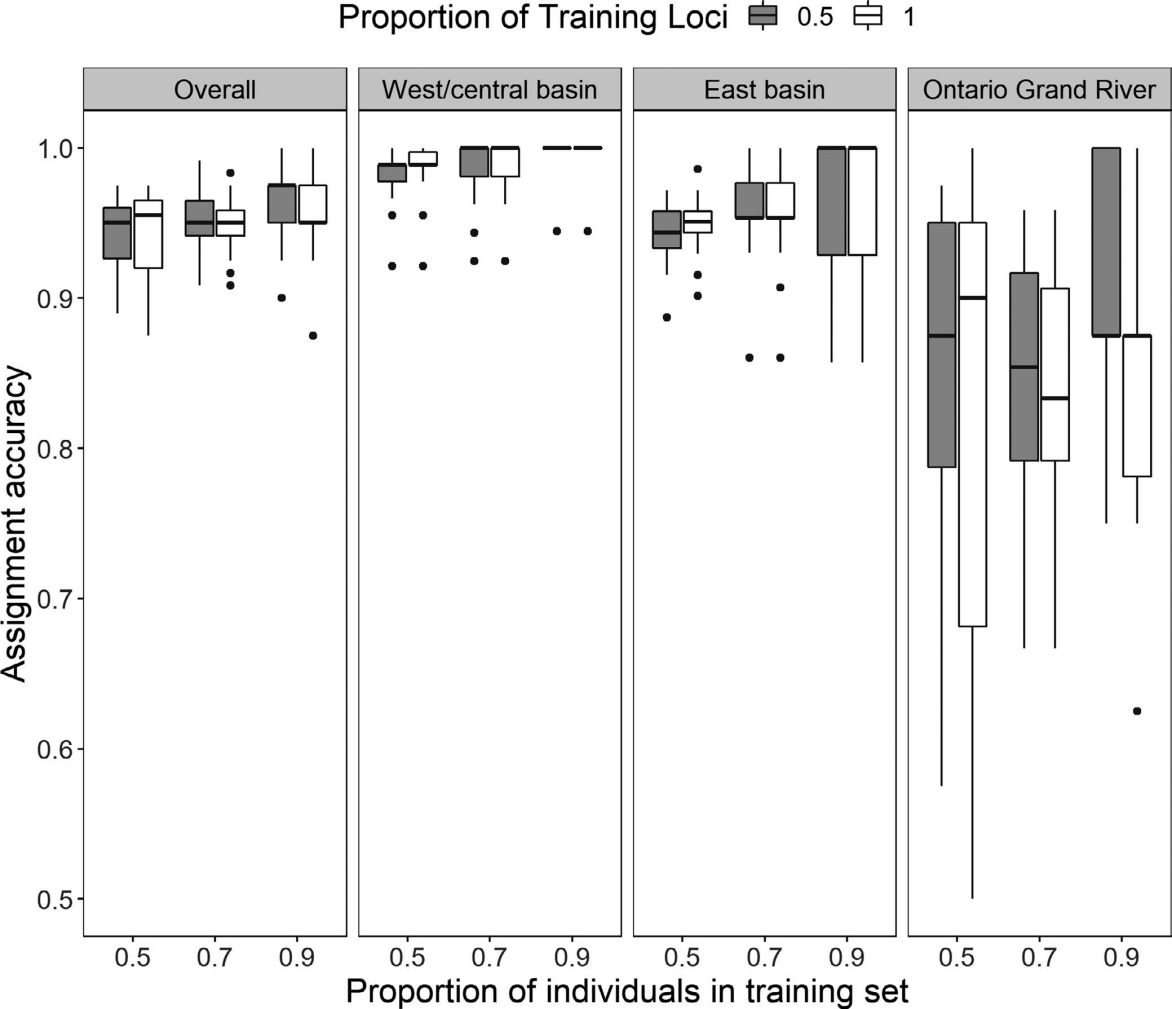
Reassignment accuracies of the three reporting groups identified based on 395 adult Walleye collected from 11 Lake Erie local spawning locations during the spawning season, 2012 – 2017. The reporting groups are comprised of fish from thefollowing: (1) the west/central basin (Maumee River, Sandusky River, Detroit River, Ohio reef complex, and Grand River, Ohio); (2) the eastern basin (Shorehaven, Bournes Beach, Van Buren Bay, Cattaraugus Creek, and Lackawanna Shore); and (3) the Grand River, Ontario. Reassignment accuracy was determined using either 0.5 or 1 proportion of training loci (gray and white bars, respectively) and a support‐vector machine algorithm (Chen, Ludsin, et al., 2020; Chen et al., 2018), with training samples for each grouping consisting of 0.5, 0.7, or 0.9 proportion of the collected individuals (chosen randomly). The remainder of individuals (0.5, 0.3, or 0.1) was used as the test (holdout) dataset to determine reassignment accuracy. Box plots portray medians (thick black line), interquartile ranges (ends of boxes), and outliers (black dots)

Reassignments of individuals to their spawning stock were low with assignPOP (<75%; Figure S1). However, when multiple stocks were combined into reporting groups that reflected the three primary clusters identified by DAPC and pairwise *F*_ST_ (western basin stocks, eastern basin stocks minus the Ontario Grand River, and the Ontario Grand River) reassignment accuracy was much higher (93%; Figure 3). The mean reassignment accuracy was near perfect for both the eastern basin and west/central basin reporting groups (96% and 99%, respectively) and slightly lower (85%) for the Ontario's Grand River reporting group. Reassignment accuracy was similar for all sets of training individuals, regardless of whether 50% or 100% of the loci were used (Figure 3). Furthermore, assessment of our baseline samples with rubias found similar reporting group accuracy as assignPOP (Figure S2), and a significant amount of variance was explained when spawning stocks were classified into these three reporting groups (AMOVA *p* = 0.01; variance between reporting groups = 1.9%, variance between populations within reporting group = 0.2%). These results gave us confidence that the west/central basin, east basin, and Ontario Grand River reporting groups could be used for mixed‐stock analyses.

### 3.2 Assignment of mixed‐stock individuals of unknown origin

Rapture sequencing of the walleye of unknown origin harvested in eastern Lake Erie's recreational and commercial fisheries produced a total of 3,331,974,311 retained reads and an average effective per‐sample coverage of 41.8 (standard deviation = 24.3). Of the 12,081 baited loci, 8482 loci passed our genotyping rate and minor allele count filters and overlapped with loci used for baseline analysis. Of the 1274 individuals analyzed, 199 of them (15%) failed to genotype in at least 50% of loci and thus were removed from analysis (Table 2). The removed samples were spread across all sampling dates (some from all 9 months) and most locations (29 of 33 grids), with the number of removed individuals being correlated with number of original samples sequenced from each grid (Pearson correlation = 0.82; *df* = 27; *p* < 0.01). Therefore, we do not believe that the removal of these samples caused any biases or was the result of any consistent laboratory error.

All individuals were assigned to either to the west/central basin or east basin reporting groups, and no individuals assigned to the Ontario's Grand River reporting group. During 2017, the total percentage of individuals of west/central basin origin was identical between individual assignments determined in assignPOP and stock mixtures estimated in rubias (west/central basin = 54%; east basin = 46%; Ontario Grand River = 0%). Owing to the variable sample sizes among collections and similarity between assignPOP and rubias, we limited our subsequent analysis to assignPOP’s individual assignments.

**FIGURE 4:**
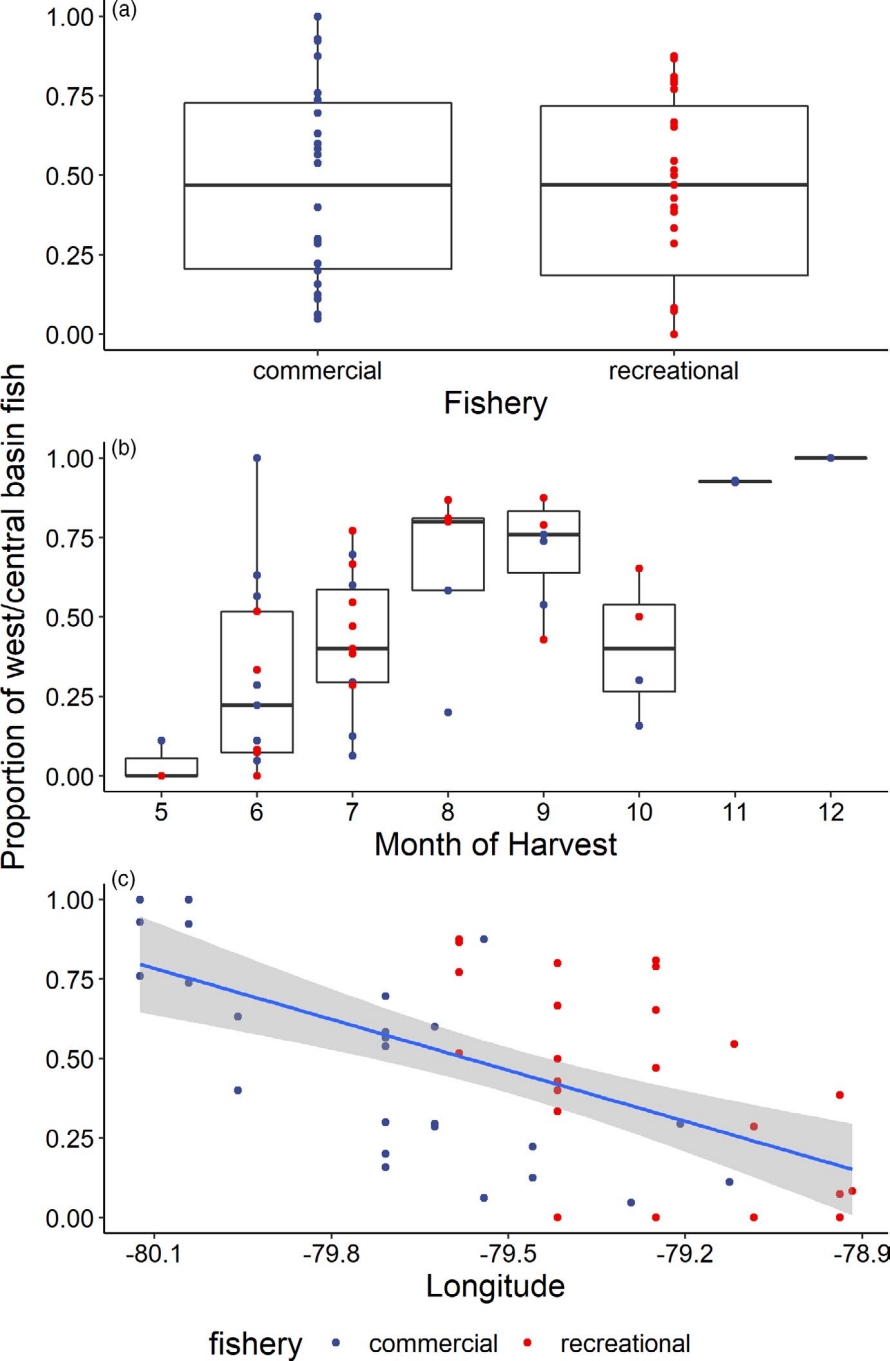
Proportion of walleye harvested in eastern Lake Erie's recreational and commercial fisheries during May through December 2017 that were assigned to the west/central basin reporting group. Proportions for both fishery types are presented for all harvested fish across all months (a), as well as for each month (b) and each harvest location (longitude; c). Each colored point represents the proportion of fish assigned to the west/central basin from a particular sampling event (harvest grid × date combination; *N* = 48). The blue line and gray background in panel C represent the least‐squares regression line from a generalized linear model and its 95% confidence interval. Note that higher sample sizes existed for core months of harvest, June–September, and thus only sampling events that contained >6 assigned individuals were included (see Table 2)

**FIGURE 5:**
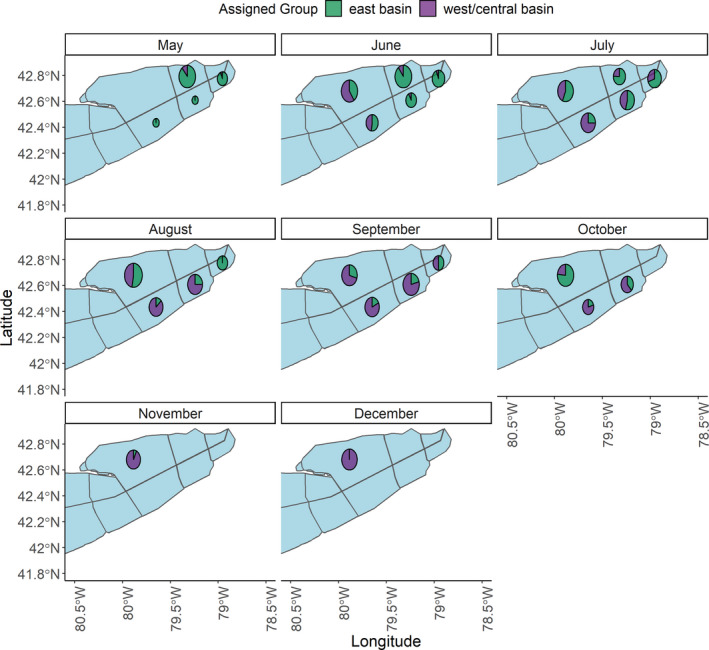
Monthly assignment of walleye harvested in eastern Lake Erie's commercial and recreational fisheries during May through December 2017 to either the west/central basin or eastern basin reporting group. The size of each pie chart in each harvest zone corresponds to log_10_ normalized mean number of fish harvested in that zone, with the pie chart being located in the harvest centroid of each rectangular harvest zone. Only two colors are shown because no harvested individuals were assigned to the Grand River (Ontario) reporting group. See Table 2 for details on samples sizes

Individual assignments were grouped by fishery type, month of capture, and longitude of capture, and the proportion of west/central basin walleye was summarized. The relative contributions from the west/central basin reporting group was near identical between fishery types (ANOVA: *F* = 0.1; *p* = 0.71; Table 2). Specifically, 51% and 49% of the walleye harvested in eastern Lake Erie's recreational and commercial fisheries, respectively, during 2017 were estimated to have originated in western/central basin (Figure 4a). While both recreational and commercial fisheries appeared to be exploiting west/central and east basin reporting groups equally, contributions to the harvest of individuals from the west/central basin varied temporally and spatially (Figure 5). During the spring, the proportion of walleye originating in the west/central basin was low (<0.10) with nearly all fish originating from eastern basin (non‐Grand River, ON) spawning stocks (Figures 4b and 5). Contributions from the west/central basin reporting group increased, however, through the summer (ANOVA: *F* = 25.1; *p* < 0.01). For example, 6% of walleye harvested in both fisheries were of west/central basin origin in May 2017, which increased to 31% by the end of June 2017 and to 50 to 75% during August/September 2017 (Figures 4b and 5; Table 2). Contributions from the west/central basin reporting group to the recreational and commercial harvest remained high throughout the summer and into the fall (July–October average percent of west/central basin origin = 57%). During November and December 2017, the fish harvested in the commercial fishery were primarily (≥93%) of western/central basin origin (Figure 4b).

Harvest composition also varied spatially during 2017 (Figures 4c and 5). In general, more easterly harvest grids had fewer individuals of west/central basin origin than more westwardly ones (ANOVA: *F* = 24.0; *p* < 0.01; Figure 4c). For example, in the most westerly harvest grids (west of 80°W), the commercial catches were dominated by west/central basin fish (≥75% of the harvest), whereas commercial catches in the most easterly grids (east of 79.5°W) were primarily of eastern basin (non‐Grand River, ON) origin (Figure 4c).

Based on examination of harvested walleye collected during July 2016–2018, contributions from the west/central basin also varied among years (Table 2). The percentage of west/central basin individuals harvested in the July 2016 recreational fishery was 20%, which was smaller than the percentage of west/central basin origin fish harvested in the recreational (54%) or commercial (38%) fisheries during 2017. However, during July 2018 the percentage of walleye of west/central basin origin in the commercial harvest was 90%, which was higher than either the commercial or recreational harvest during July in the previous two years (Table 2).

### 3.3 Predicting contributions from the west/central basins

To better predict proportional contributions of western/central basin spawning stocks to eastern Lake Erie's recreational and commercial fisheries, we sought to use AICc and an all‐subsets approach to evaluate a set of general linear models. Two variables (fishery type and mean total length) were not significantly related to the proportion of west/central basin fish (ANOVA: both *r* ≤ 0.1, both *p* ≥ 0.05), we removed them prior to AIC_c_ analysis. All other variables (longitude, latitude, and month) explained a significant amount of the variance and were included in our AIC_c_ assessment. Our modeling supported the notion that the proportional contribution from the west/central basin reporting group to the eastern basin fisheries in 2017 varied both seasonally and spatially. The candidate model with the highest level of support in our AIC_c_ analysis contained all three remaining potential predictors (Table 4). This model (coefficients: longitude = −0.37, latitude = −0.74, month = 0.05, intercept = 2.8; *df* = 47) had an evidence ratio that indicated it was 10 times more likely than the next best model and the next best model had a ∆AIC_c_ value of 4.52, indicating that it was more parsimonious than any of the others (Burnham & Anderson, 2004). Longitude appeared to be the most important predictor of proportional contributions of western/central basin spawning stocks to the east basin harvest, with contributions from this reporting group declining from west to east based AIC_c_ weight of the top two models (Figure 4c). However, date and latitude also were important, with proportional contributions of west/central basin reporting group decreasing from north to south and from spring through fall.

**TABLE 4:** Statistics from comparisons of general linear models used to describe the proportion of western basin walleye (PropWB) harvested in eastern Lake Erie's recreational and commercial fisheries during May through December 2017 as a function of longitude of each harvest grid (lon), latitude of each harvest grid (lat), and the month of harvest (month)

Model	*K*	AIC_c_	∆AIC_c_	AIC_c_Wt	LL	ER
**PropWB = lon + lat + month**	**5**	**−5.54**	—	**0.869**	**8.47**	**—**
PropWB = lon + lat	4	−0.99	4.52	0.091	4.96	10
PropWB = lon + month	4	1.28	6.79	0.029	3.83	32
PropWB = lat + month	4	3.64	9.15	0.009	2.65	103
PropWB = lon	3	7.61	13.12	0.001	−0.53	788
PropWB = month	3	8.33	13.84	0.001	−0.89	1130
PropWB = lat	3	19.11	24.62	<0.001	−6.28	247,246
ProWB = 1	3	25.78	31.29	<0.001	−10.78	7,309,885

Note

The number of estimated parameters for each model (*K*), Akaike's information criterion for small sample sizes (AIC_c_), change in information criterion between sequential models (∆AIC_c_), AIC_c_ weight (AIC_c_Wt), log likelihood (LL), and evidence ratio (ER) is provided. The most parsimonious model is in bold‐face text.

## 4 DISCUSSION

Our study used a sequencing‐based genetic Rapture panel to reveal weak, yet sufficient, population structure and to reliably quantify the relative contributions of distinct spawning stocks to the harvest in a mixed‐stock fishery. Specifically, using microhaplotypes taken from mature walleye collected at 11 Lake Erie spawning locations during the spawning season, we developed classification functions that could assign walleye of unknown origin to a putative spawning region with a high degree of accuracy. Our findings supported our expectation that walleye from the western basin contribute substantially to the eastern basin fisheries for much of the year. Even so, we found seasonal and spatial variation in the proportion of walleye originating from the west/central basin reporting group. This result indicates that the smaller eastern basin stocks (except for the Grand River, Ontario) comprise a large portion of harvest during certain times (e.g., during the spring) and in certain areas of the eastern basin (e.g., locations east of 79.5°W). In addition to providing critical information on stock‐specific harvest of walleye in the eastern basin of Lake Erie that can benefit fishery management, our study represents one of the first uses of Rapture data for mixed‐stock assignment in fisheries (but see Carrier et al., 2020) and highlights the value of genomic approaches for management and conservation applications involving species with weak population structure.

### 4.1 Population structure and reassignment accuracy

Although our Rapture panel could not reliably discern individual spawning stocks within each basin, we were able to use it to discriminate among three reporting groups (i.e., west/central basin, east basin, and Ontario Grand River) with high reassignment accuracy (93%). Previous attempts at differentiating Lake Erie stocks of walleye using microsatellite loci (Brenden et al., 2015; Stepien et al., 2012) or mitochondrial DNA (mtDNA) haplotypes (Gatt et al., 2004; Haponski et al., 2014; Stepien & Faber, 1998) similarly identified genetic differences between basins, but also could not consistently or reliably assign individuals to independent stocks (20%–87% assignment accuracy).

Our success in developing reliable assignment functions emanated from three sources. First, in contrast to earlier studies (Stepien et al., 2012; Strange & Stepien, 2007), we aggregated spawning stocks with low genetic differentiation and included more stocks into our baseline (Brenden et al., 2015; Chen, Euclide, et al., 2020). Doing so helped us to identify a robust set of reporting groups, albeit at a coarser spatial scale. Second, we used high‐throughput sequencing to genotype thousands of microhaplotype loci, instead of using a microsatellite panel or mtDNA, which increased our statistical power and identified useful genetic variation among stocks that could be used to assign harvested individuals of unknown origin to a natal source (i.e., area of origin). Third, extensive preliminary tests of assignment accuracy in both assignPOP and rubias provided us with high confidence in individual assignments. These individual assignments of walleye facilitated the detection of fine‐scale patterns in stock‐specific harvest, which will have direct implications for the sustainable management of this ecologically and economically important population (Hatch et al., 1987; Kayle et al., 2015).

### 4.2 Stock contributions to eastern basin fisheries

The contribution of west/central basin stocks to eastern basin fisheries was predicted to be large based on previous estimates suggesting that greater than 90% of the harvest in the eastern basin comes from western basin stocks (Walleye Task Group, 2018; Zhao et al., 2011). However, previous estimates were unable to quantify seasonal, annual, and spatial variation in harvest contributions. Here, we show that while seasonal eastward migrations of walleye from west/central basin spawning stocks do contribute substantially to walleye harvest in the eastern basin, contributions can be highly variable among years, seasons, and locations.

Although general seasonal patterns in harvest contributions of west/central basin stocks were observed, the level of spatiotemporal variability indicates that harvest pressure on less productive eastern basin stocks may be difficult to predict. Contributions of west/central basin walleye in the eastern basin generally increased rapidly following the spawning period in April. By early summer (June–July) west/central basin walleye contributed substantially to the harvest, and by August made up the majority of genotyped fish in both the recreational and commercial fisheries. These findings are in accordance with walleye movement patterns identified through acoustic telemetry (Matley et al., 2020). However, the contribution of west/central basin fish varied greatly from year‐to‐year even during periods of high harvest (20% in 2016 to 90% in 2018). Results also indicated that stocks do not completely mix within the eastern basin but instead show a longitudinal gradient, with the west/central basin contribution decreasing from west to east. This trend was especially clear in June and July, during which west/central origin fish made up 50%–75% of assigned individuals in westerly samples and only about 25% in easterly samples.

The magnitude and extent of annual migrations have been hypothesized to be associated with western basin population size (Zhao et al., 2011) water temperature (Raby et al., 2018), and prey availability (Kershner et al., 1999). Therefore, interannual variation in these factors likely drives walleye distribution and harvest dynamics throughout Lake Erie (Dippold, Adams, et al., 2020). In years when eastward migration is either delayed or limited, eastern basin stocks may be exploited to a higher degree (Dippold, Aloysius, et al., 2020). Over longer periods of time, exploitation of individual stocks may also vary with their relative abundance owing to differences in productivity and recruitment success between eastern and western basin stocks. Behavioral differences among stocks might also influence contributions to the mixed fishery. For example, there is evidence that walleye from Ontario's Grand River do not mix evenly with walleye from the west/central and east basin reporting groups (Jackson et al., 2003; Matley et al., 2020), and no fish in our mixed‐stock analysis were assigned to the Ontario Grand River despite previous evidence of Grand River walleye contributing to the eastern basin harvest (Jackson et al., 2003). Further understanding of walleye migration patterns and their environmental predictors may inform harvest quotas in Lake Erie, as they have in other fisheries (Bradbury et al., 2016; Shaklee et al., 1999; Vähä et al., 2011).

One of the most surprising findings of this study was that eastern basin walleye stocks have a larger influence on eastern basin fisheries than previously thought and likely contribute substantially to harvest from year‐to‐year. In particular, fisheries in the eastern‐most portion of the eastern basin appear to be supported primarily by walleye of eastern basin origin, and therefore declines in eastern basin stocks could have substantial impacts on fishing opportunities in this region. The spatiotemporal variability of west/central basin walleye contributions to eastern basin harvest supports the hypothesis that the Lake Erie walleye population functions as a portfolio, whereby both eastern and western basin spawning stocks contribute to the overall stability of the lake‐wide population (DuFour et al., 2015). While western basin stocks produce the majority of walleye biomass in Lake Erie, loss or reduction of smaller eastern basin stocks could have a large impact on fishing and harvest opportunities in the eastern basin, and on the diversity and resiliency of the portfolio. These results indicate that periodic reassessments of stock contributions to the harvest are likely necessary to characterize longer‐term spatiotemporal variation in relative stock contributions and to inform management decisions. Prior to development of our Rapture panel, repeated mixed‐stock assessments of walleye were largely seen as unfeasible or too imprecise to merit the investment. Now that a methodology capable of conducting high‐accuracy mixed‐stock assignment has been established, the likelihood that re‐assessments will take place is greatly increased.

### 4.3 Implications for inland fisheries conservation and management

Many inland fisheries already experience high spatiotemporal variability in harvest, behavior, and recruitment success (e.g., Page et al., 2003; Taabu‐Munyaho et al., 2014; Thorstensen et al., 2020). Continued climate warming is predicted to interrupt natural patterns of recruitment for many species, including walleye in western Lake Erie (Brander, 2007; Dippold, Aloysius, et al., 2020). The ability to manage populations like investment portfolios, whereby agencies invest in the protection of multiple spawning stocks could promote stability within these ecosystems and the fisheries that they support (e.g., Schindler et al., 2015; Waples et al., 2020). However, this strategy depends on the ability to distinguish among population components. The development and use of new molecular tools provide opportunities to incorporate portfolio theory into the management in new fish populations that could benefit from this strategy.

Without a doubt, our ability to understand stock‐specific production and harvest in Lake Erie will depend on the continued use of genomic methods. The increased diagnostic power of our Rapture panel made it possible to make precise estimates stock structure. Indeed, when genetic differentiation is low, as is the case in Lake Erie walleye (Chen, Ludsin, et al., 2020; this study) having a high number of genetic markers becomes more important for high assignment accuracy (Benestan et al., 2015; Larson et al., 2014; Waples & Gaggiotti, 2006). The high assignment accuracy allowed us to go beyond mixture analysis, which is generally considered to be more accurate than individual assignments when genetic structure is low (Manel et al., 2005) and investigate fine‐scale variation in harvest proportions. Genotyping‐by‐sequencing methods, like Rapture, offer a feasible way to obtain high diagnostic power in a nonmodel species and can provide reliable mixed‐stock estimates, even when population components (e.g., local spawning populations) are weakly differentiated. Such approaches could be used in other ecosystems to offer conservation and management agencies the ability to quantify the relative contributions of local spawning populations to larger population they support.

With the promise that genomic tools hold, we are optimistic that molecular studies of exploited freshwater populations can begin to be used more consistently to monitor contemporary changes in population structure and microevolution in response to anthropogenic change. Collecting the data necessary for mixed‐stock analysis can be problematic for data‐poor fisheries that lack established molecular resources such as SNP or microsatellite marker panels. Our approach does not require prior‐knowledge about the genetic background of a population and therefore could provide a means to conduct mixed‐stock assessments in most ecosystems (Allan et al., 2005; Irvine et al., 2019). In this way, we are confident that the continued use of genomic approaches, like the one demonstrated herein, can aid efforts to unravel the complexities associated with threatened or exploited populations that are supported by multiple local breeding populations such that they can remain sustainable both now and in the face of future ecosystem change.

## CONFLICT OF INTEREST

There is no conflict of interest declared in this article.

## Supporting information

Figures S1‐S2

Table S1

Table S2

## Data Availability

The data that support the findings of this study are openly available in Geome at https://geome‐db.org/workbench/project‐overview?projectId=190 and Dryad at https://doi.org/10.5061/dryad.4b8gthtb2. All raw sequence files are available via the NCBI short read archive under the BioProject ID’s: PRJNA699858, PRJNA698671, PRJNA702563, PRJNA702508, PRJNA702065, PRJNA702136.
