# Supplementary material for: Mixed‐stock analysis using Rapture genotyping to evaluate stock‐specific exploitation of a walleye population despite weak genetic structure
Source: Evol Appl. 2021 Mar 30;14(5):1403–20. doi: 10.1111/eva.13209 (PMC8127713; doi:10.1111/eva.13209)
Supplement: Supplementary file 1 — Figures S1‐S2 [file EVA-14-1403-s002.docx]

Figure S1: Reassignment accuracies of 11 spawning stocks identified based on 395 adult Walleye collected from 11 Lake Erie spawning sites between 2012–2017 calculated in assignPOP: West/Central Basin – Maumee River (MA), Sandusky River (SA), Detroit River (DE), the Ohio reef complex (RE), Ohio Grand River (OHG), Ontario Grand River– Ontario Grand River (ONG), East Basin – Shorehaven (SH), Bourus Beach (BB), Van Buren Bay (VB), Cattaraugus Creek (CC), Smokes Creek (SC). Reassignment accuracy was determined using either 0.5 or 1 proportion of training loci (colors) and a support-vector machine algorithm, with training samples for each grouping consisting of 0.5, 0.7, or 0.9 proportion of the collected individuals (chosen randomly). The remainder of individuals (0.5, 0.3, or 0.1) was used as the test (holdout) data set to determine reassignment accuracy. Box plots portray medians (thick black line), interquartile ranges (ends of boxes), and outliers (black dots).


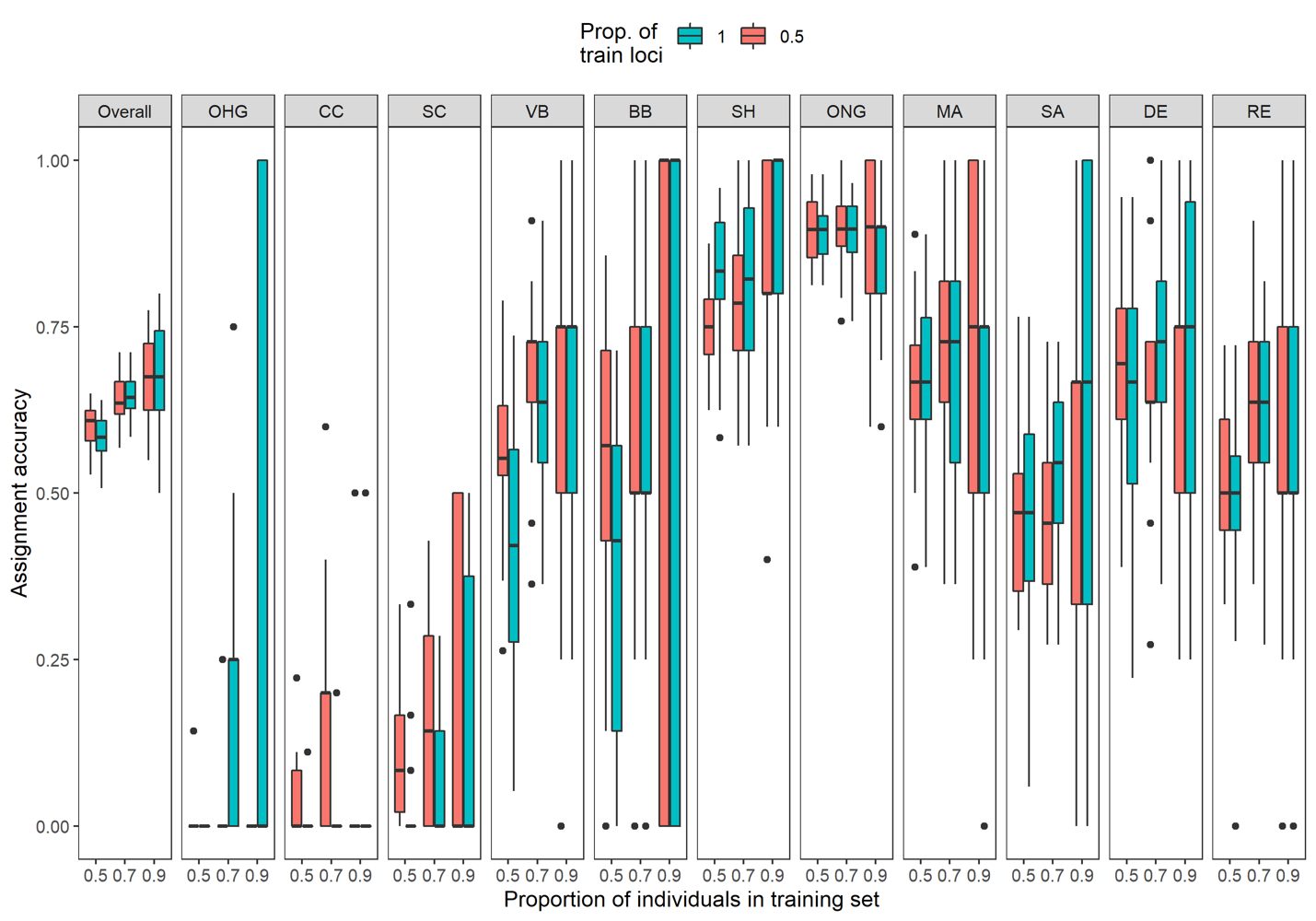


Figure S2: Summary of rubias assignment accuracy of three Lake Erie reporting groups Ontario Grand River (ONG), west/central basin (West_Basin) and east basin (East_Basin). Mean posterior mixing proportions of 100% mixtures for each reporting group across the first 25 iterations (top) and distribution of individual posterior reporting group proportion for simulated collections from 100% mixtures of each reporting group.


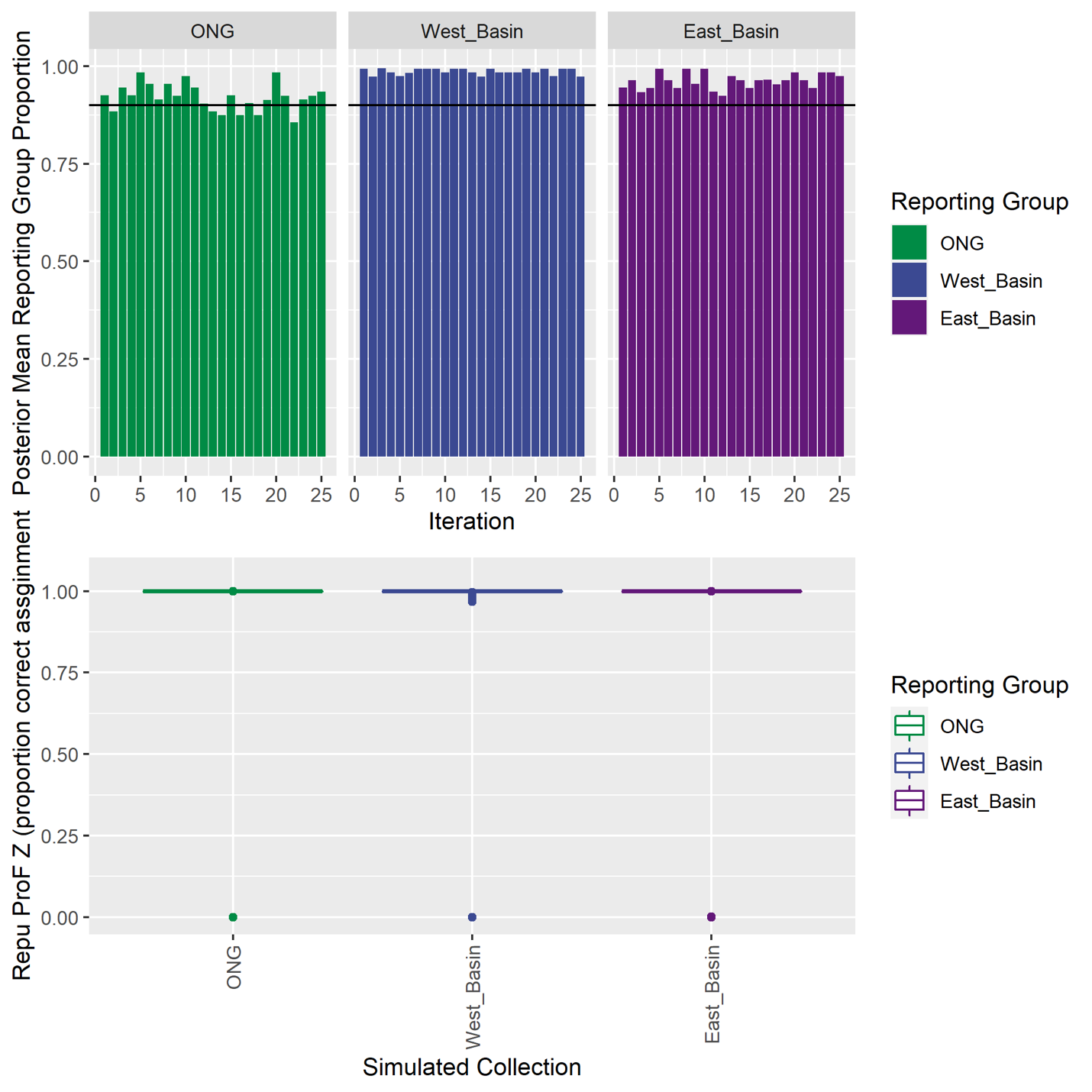


Suppl. Table 1: Metadata collected for all walleye of unknown origin that were collected in eastern Lake Erie’s commercial and recreational fisheries and used in our mixed-stock analyses. Walleye were collected during 2016 – 2018 by the New York State Department of Environmental Conservation and Ontario Ministry of Natural Resources and Forestry. Also included is the reporting group (west/central basin or eastern basin) to which each individual was assigned using assignPOP.

Suppl. Table 2: Locus-specific diversity estimates (Locus ID, number of alleles, effective number of alleles, observed heterozygosity, inbreeding coefficient (G_IS_), and genetic distance estimate (G_ST_) for the 8,482 microhaplotypes used for individual assignments of walleye collected at spawning locations throughout Lake Erie during the 2012 – 2018 spawning seasons.

Suppl. Fig. 1: Reassignment accuracies from assignPOP of 11 spawning stocks identified based on 395 adult walleye collected from 11 Lake Erie spawning locations during the 2012–2017 spawning seasons. The spawning stocks consisted of individuals collected from the western and central basins (Maumee River [MA], Sandusky River [SA], Detroit River [DE], the Ohio reef complex [RE], and Ohio Grand River [OHG]), the eastern basin (Shorehaven [SH], Bourus Beach [BB], Van Buren Bay [VB], Cattaraugus Creek [CC], and Smokes Creek [SC]), and the Ontario Grand River (ONG). Reassignment accuracy to each spawning stock was determined using a support-vector machine algorithm (Chen et al. 2018, 2020a) using different proportions (0.5 or 1.0) of the training loci (colors of boxes) and different proportions of individuals collected at each location used in the training samples (0.5, 0.7, or 0.9 of the collected individuals, chosen randomly). The remainder of individuals (0.5, 0.3, or 0.1) was used as the test (holdout) dataset to determine reassignment accuracy. Box plots portray medians (thick black line), interquartile ranges (ends of boxes), and outliers (black dots).

Suppl. Fig. 2: Reassignment accuracies from rubias of three reporting groups based on 395 adult walleye collected from 11 Lake Erie spawning locations during the 2012–2018 spawning seasons. The three reporting groups were the Ontario Grand River (ONG), west/central basin (West_Basin), and eastern basin (East_Basin). Mean posterior mixing proportions of 100% mixtures for each reporting group across the first 25 iterations (top) and distribution of individual posterior reporting group proportion for simulated collections from 100% mixtures of each reporting group.
